# Improving Blood Pressure Control and Tobacco Use Cessation Intervention In Primary Care: Protocol for the Alabama Cardiovascular Cooperative Heart Health Improvement Project

**DOI:** 10.2196/63685

**Published:** 2024-12-20

**Authors:** Kathryn Foti, Demetria Hubbard, Kimberly A Smith, Larry Hearld, Joshua Richman, Trudi Horton, Sharon Parker, Dodey Roughton, Macie Craft, Stephen A Clarkson, Elizabeth A Jackson, Andrea L Cherrington

**Affiliations:** 1 Department of Epidemiology University of Alabama at Birmingham Birmingham, AL United States; 2 Department of Family and Community Medicine University of Alabama at Birmingham Heersink School of Medicine Birmingham, AL United States; 3 Department of Health Services Administration University of Alabama at Birmingham School of Health Professions Birmingham, AL United States; 4 Department of Surgery University of Alabama at Birmingham Heersink School of Medicine Birmingham, AL United States; 5 Division of General Internal Medicine and Population Science Department of Medicine University of Alabama at Birmingham Heersink School of Medicine Birmingham, AL United States; 6 Alabama Primary Health Care Association Montgomery, AL United States; 7 Division of Cardiovascular Disease Department of Medicine University of Alabama at Birmingham Heersink School of Medicine Birmingham, AL United States

**Keywords:** hypertension, primary care, quality improvement, tobacco use, smoking cessation, healthcare quality, quality of care, risk modification, cardiovascular disease prevention

## Abstract

**Background:**

Alabama has the second highest rate of cardiovascular disease (CVD) mortality of any US state and a high prevalence of CVD risk factors such as hypertension, diabetes, obesity, and smoking. Within the state, there are disparities in CVD outcomes and risk factors by race or ethnicity and geography. Many primary care practices do not have the capacity for full-scale quality improvement (QI) initiatives. The Alabama Cardiovascular Cooperative (ALCC), which includes academic and community stakeholders, was formed to support primary care practices to implement QI initiatives to improve cardiovascular health. The ALCC is implementing a Heart Health Improvement Project (HHIP) in primary care practices with suboptimal rates of blood pressure (BP) control and tobacco use screening.

**Objective:**

The study aimed to support primary care practices to increase BP control among adults with hypertension and increase rates of tobacco use screening and cessation intervention.

**Methods:**

We are using a type 1 hybrid design to test the effects of the HHIP on BP control among adults with hypertension and tobacco use screening and cessation intervention, while collecting information on implementation. Primary care practices were recruited through existing practice networks and additional electronic and in-person outreach. To ensure participation from a broad range of clinics, we required at least 50% of practices to be Federally Qualified Health Centers or look-alikes and to include representation from practices in rural areas. At baseline, we collected information about practice characteristics and preintervention rates of BP control and tobacco use screening and cessation intervention. The QI intervention includes quarterly activities conducted over a 12-month period. The HHIP uses a multipronged approach to QI, including practice facilitation and technical assistance, on-site and e-learning, and improvement through data transparency. We will conduct a pre-post analysis to estimate the effects of the HHIP and whether there is an enduring change in outcomes after the 12 months of HHIP activities beyond what would be expected due to secular trends.

**Results:**

Practice recruitment took place between April 2021 and October 2022. After contacting 417 primary care practices, 51 were enrolled, including 28 Federally Qualified Health Centers or look-alikes; 47 practices implemented the HHIP. Among 45 practices that completed the baseline survey, 11 (24%) were solo practices, while 28 (62%) had 1-5 clinicians, and 6 (13%) had 6 or more clinicians. The median number of patient visits per year was 5819 (IQR 3707.3-8630.5). Practices had been in operation for a mean of 19.2 (SD 13.0) years. At baseline, the mean BP control rate was 49.6% and the rate of tobacco use screening and cessation intervention was 67.4%.

**Conclusions:**

If successful, the ALCC and HHIP may improve the implementation of evidence-based guidelines in primary care and, subsequently, cardiovascular health and health equity in the state of Alabama.

**International Registered Report Identifier (IRRID):**

DERR1-10.2196/63685

## Introduction

In the United States, cardiovascular disease (CVD) mortality declined dramatically beginning in the 1960s [1,2]. However, the decline has stalled since the 2010s, and national trends mask uneven progress by race or ethnicity and geography [2-5]. As a result, inequities in cardiovascular outcomes persist, and in some cases have widened, across racial, socioeconomic, and geographic lines [5,6].

Alabama has the second-highest rate of CVD mortality of any state in the nation [7]. The rate is even higher among non-Hispanic Black versus non-Hispanic White populations in the state and higher in rural versus urban counties [8,9]. Alabama also has a high prevalence of CVD risk factors such as hypertension, diabetes, obesity, and smoking [10,11]. The impact of these risk factors on health outcomes may be exacerbated by a lack of access to primary care. Compared with the top performing states (90th percentile), which have an average of 1 primary care physician per 1030 residents, Alabama has 1 primary care physician per 1540 residents, ranging from 1:930 to 1:10,080 depending on the county [12]. All but 5 of Alabama’s 67 counties are deemed Primary Care Health Professional Shortage Areas by the Health Resources and Services Administration [13]. Few primary care practices have sufficient administrative support or the funding needed to facilitate full-scale quality improvement (QI) efforts.

To address these challenges, several organizations have provided services to support QI initiatives across the state, including the Alabama Primary Health Care Association (APHCA), QSource, and the Alabama Department of Public Health. In 2021, these organizations came together with academic partners from the Auburn University School of Pharmacy and the University of Alabama at Birmingham (UAB) Heersink School of Medicine to form the Alabama Cardiovascular Cooperative (ALCC) to support primary care practices across the state to implement QI initiatives to improve cardiovascular health. This paper describes the protocol for the ALCC’s Heart Health Improvement Project (HHIP), a QI project implemented in 47 primary care practices to improve the assessment and management of cardiovascular risk factors, specifically hypertension and tobacco use. The HHIP will use a type 1 hybrid design [14] to test the effects of the HHIP on clinical outcomes at the practice level, including blood pressure (BP) control among adults with hypertension and tobacco use screening and cessation intervention, while collecting information on implementation.

## Methods

### ALCC Overview

**Figure 1:**
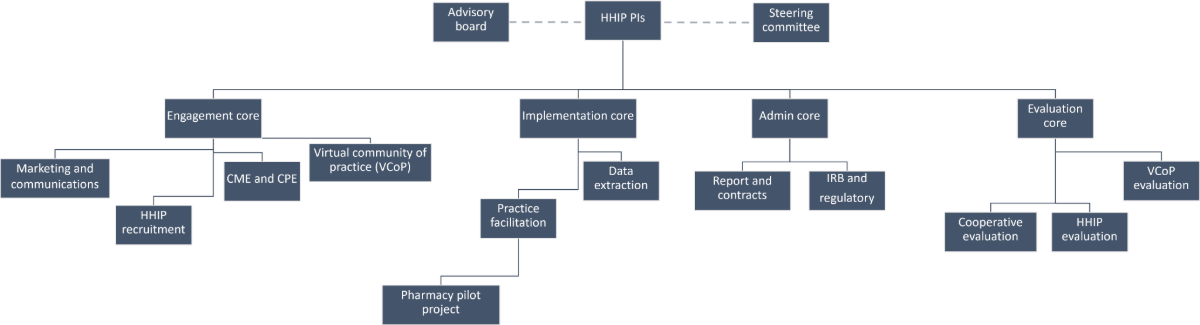
Alabama Cardiovascular Cooperative Organizational Structure. CME: continuing medical education; CPE: continuing pharmacy education; HHIP: Heart Health Improvement Project; IRB: institutional review board; PI: principal investigator; VCoP: virtual community of practice.

The ALCC was formed to coordinate statewide efforts to reduce cardiovascular risk and disparities among individuals across the state of Alabama by supporting primary care providers, especially those in the most underserved areas of the state (Figure 1). Each of the organizations that is part of the ALCC has long-standing commitments to improving health outcomes in Alabama, broad statewide reach, and a shared belief that a coordinated effort to support primary care–based initiatives to improve cardiovascular health in Alabama could prove transformative. An Advisory Board, which includes representation from key stakeholders from multiple sectors across the state, helps inform the Cooperative’s activities and sustainability plans through quarterly meetings and ad hoc communication. The Cooperative serves as a convener of stakeholders and maintains a repository of ongoing CVD-related initiatives and resources. The Cooperative is also implementing the HHIP in 47 clinics to improve rates of BP control and tobacco use intervention.

**Figure 2:**
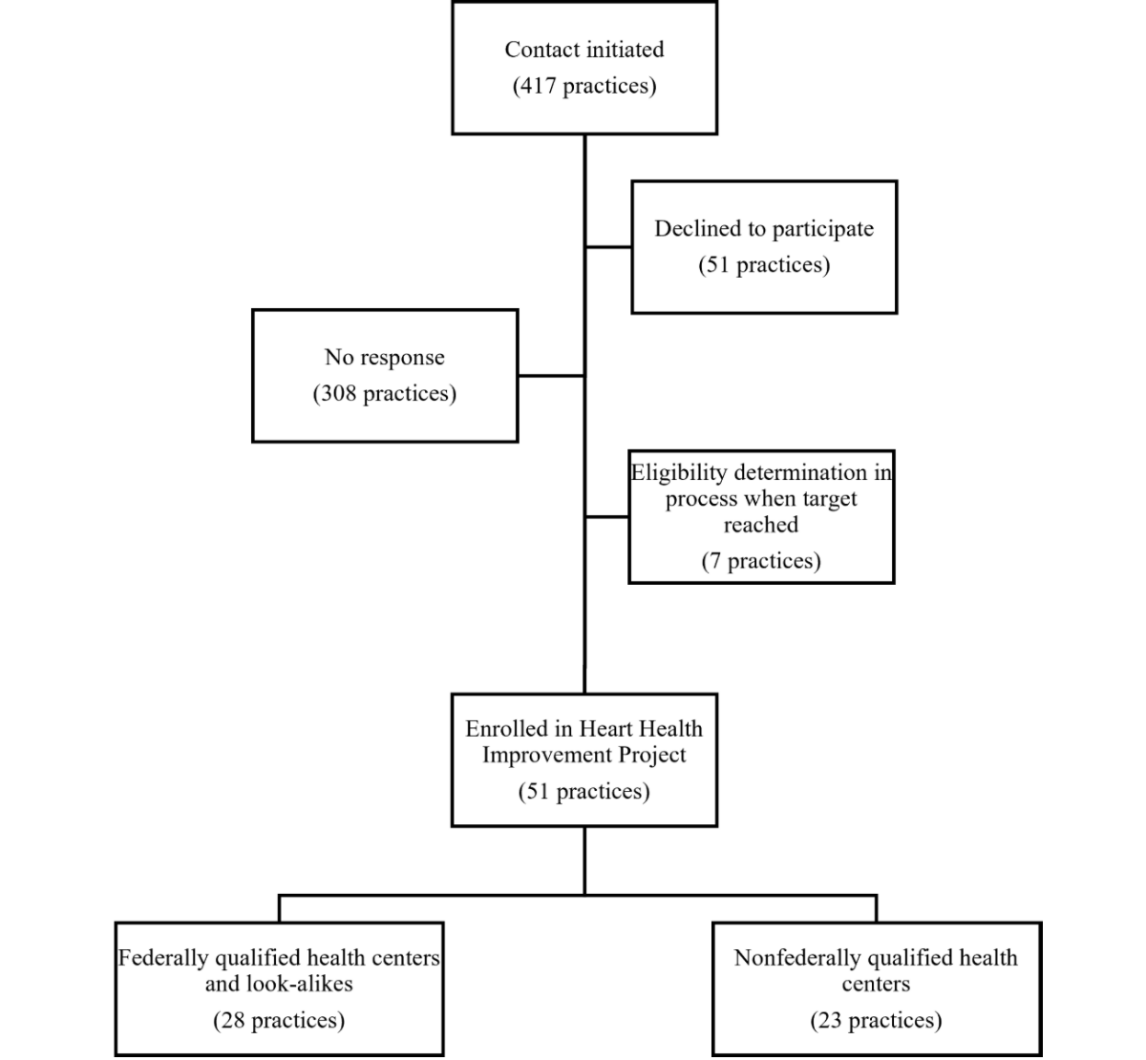
Heart Health Improvement Project primary care practice recruitment.

### HHIP Overview

#### Approach to QI

The HHIP uses a multipronged approach to QI, including practice facilitation and technical assistance, on-site and e-learning, and improvement through data transparency. These strategies, described in further detail in this paper, were selected by the study investigators based on a review of patient-centered outcomes research. The HHIP is designed to build internal QI capacity and increase the implementation of evidence-based guideline recommendations for screening and treatment of cardiovascular risk factors. This project builds on the strengths and the previous and ongoing efforts of all Cooperative partners, such as (1) the Southeastern Collaboration to Improve Blood Pressure, a recent trial assessing the use of practice facilitation to improve BP control among adults with hypertension in primary care (UAB) [15]; (2) the Blood Pressure Self-Management Initiative (Alabama Department of Public Health); (3) expertise in promotion of best practices for pharmacotherapy (Auburn); as well as (4) ongoing QI initiatives in primary care (QSource and APHCA).

#### Study Design

The HHIP was initially designed as a “stepped wedge” in stages with groups of 10 practices randomly selected to begin the intervention each quarter. However, delays in practice recruitment did not allow for the randomization of practices by quarter, so we instead used a nonrandomized, pre-post design with primary care clinics as the unit of observation. Despite the design change, quantitative analysis of the effect of HHIP on measures of hypertension and smoking cessation will follow the general framework for the analysis of a stepped-wedge study. This includes using preintervention data from all practices to estimate secular trends—changes expected to occur without intervention, as well as estimating effects from the HHIP, and whether there is an enduring change in outcomes after the year of HHIP activities beyond what would be expected due to secular trends. Models will include random effects for sites and for patients to account for multiple patients nested within sites and the potential for multiple observations per patient. Secondary clinic-level analyses will use data aggregated each quarter by clinic.

#### Practice Recruitment

The Cooperative used several methods of recruitment including existing organization communication channels to recruit primary care practices for inclusion in the HHIP. First, we reached out to clinics in existing networks through the Alabama Practice-Based Research Network, the APHCA, and QSource, leveraging existing relationships for introductions. In addition, we created flyers and promotional materials to send to clinics through email and fax. We also conducted Google searches for clinics in designated areas to find additional practices to recruit while we were visiting enrolled sites. To ensure we reached a broad range of clinics, we aimed for at least 50% of our clinics to be Federally Qualified Health Centers (FQHCs) or look-alikes, which have an important role in increasing access to primary care in medically underserved areas, including rural communities [16,17].

For each practice that expressed interest in participation, we conducted an informational recruitment meeting to further explain the HHIP objectives and procedures. During this initial meeting, interested clinics completed readiness assessments using standardized questionnaires [18,19] and signed an informal letter of agreement to participate in the project. Following the initial meeting, the questionnaire scores and clinic information (eg, anecdotal patient demographics and clinic estimation of BP control rate) were shared with the Cooperative, and practices were voted on to be enrolled in the project. Once a practice was enrolled, a lead practice facilitator was assigned.

Practice facilitators scheduled and conducted a kick-off meeting for the practices that were enrolled in the HHIP. This kick-off meeting served as the start of the intervention where practices received additional information about practice facilitation, completed an “About Your Practice” survey, and initiated processes required to extract baseline data. Enrolled practices were also asked to identify a practice champion as part of the enrollment process. Practice champions are integral to the successful adoption of innovations within a practice, defined as “individuals who dedicate themselves to supporting, marketing, and driving through an implementation, overcoming indifference or resistance that the intervention may provoke in an organization” [20]. The practice champion worked with the practice facilitator to schedule monthly touchpoints throughout the intervention period to implement and track the progress of quarterly QI activities. Clinics were compensated US $1500 for their time and participation in the intervention.

#### Intervention

##### Practice Facilitation

The HHIP implementation strategies included practice facilitation and technical assistance, on-site and e-learning, and improvement through data transparency, with practice facilitation serving as the foundation of the intervention. Practice facilitation is a flexible, tailored service aimed at assisting practices in implementing evidence-based care guidelines, thereby improving care quality and patient outcomes. Practice facilitators enable rather than direct QI activities, thereby building a practice’s internal capacity for adapting clinical evidence to their practice context [21].

The practice facilitation intervention is built on the key driver model for care implementation [22] that was developed as part of the Improving Performance in Practice initiative and whose drivers focus on 4 components of Wagner’s Chronic Care Model—delivery system design, decision support, clinical information systems, and self-management support [22-24]. As shown in Table 1, the facilitator guides the practices in targeting these key drivers to encourage practice-wide improvements in using registries, delivering planned care, using standardized care processes, and providing effective self-management support.

**Table 1:** Key drivers and relationship to chronic care model.

Key drivers	Examples of QI^a^ activities	Chronic care model components
Implement registry	Determine staff workflow to support registry Populate registry Maintain registry	Clinical information systems Delivery system design
Use templates for planned care	Determine staff workflow to support template Use template with all patients Update registry each time template is used and monitor its use	Decision support Delivery system design
Employ protocols	Select and customize evidence-based protocols Determine staff workflow to support protocol Use protocol with all patients Monitor use of protocols	Decision support Delivery system design
Provide self-management support	Select patient education materials Determine staff workflow to support patient self-management Collaboratively set goals with patients Monitor patient progress toward goals Link patients with community resources	Self-management support Community resources

^a^QI: quality improvement.

The HHIP practice facilitators are used by organizations in the ALCC. Each of the facilitators received dedicated training delivered by HealthTeamWorks and the Agency for Healthcare Research and Quality Primary Care Practice Facilitation Curriculum to support practices to implement a change package [25]. The practice facilitators work with practices over a 1-year period, with at least monthly in-person (or by teleconference if necessary) visits and biweekly teleconferences with the practice champion and, additionally or instead, the QI team. At the start of the intervention, practices complete a self-assessment to measure quality indicators and identify the current level of QI implementation using the Key Drivers Implementation Scale (Multimedia Appendix 1) [26]. The self-assessments provide baseline data on practice performance as well as baseline data on practice capacity for QI (eg, generating reliable, valid performance data; seeking and integrating evidence-based guidelines into patient care; creating patient panels and using continuous QI processes involving care teams to identify and assist at-risk patients, and engaging patients and families to provide effective self-management support).

Following baseline assessments, practice facilitators guide practices to implement QI activities designed to achieve progress in each key driver area. These include activities such as using the electronic health record (EHR) to enable data collection, creating registries to track the implementation of evidence-based care, constructing a QI dashboard to enable ongoing reporting of process and outcome measures, establishing team-based care processes, and engaging patients and families to optimize their self-management goals. Practice facilitators encourage practices to conduct quarterly QI initiatives, beginning with a focus on hypertension control and moving to tobacco use cessation in at least 1 subsequent quarter.

##### e-Learning

The ALCC also uses e-learning to provide pragmatic guidance on cardiovascular risk reduction in primary care. As approved by Continuing Medical Education (UAB) and Continuing Pharmacy Education (Auburn) providers with access to cardiovascular experts, we offer accredited hours through asynchronous e-learning sessions for physicians and midlevel providers. Based on initial feedback regarding sessions, we also created sessions specific for staff members. Working closely with the ALCC, the UAB Division of Continuing Medical Education designed an initiative that is grounded in blended learning theory and creates a learning environment that fosters the adoption of new practices. The educational intervention focuses on topics critical to accurate patient risk assessment and appropriate guideline-concordant treatment of individuals at risk for CVD. Initial topics were identified by the Cooperative’s Steering Committee.

A provider needs assessment was conducted with attendees at an Alabama Academy of Family Physicians meeting, which further informed the educational topics, preferred mode of delivery, as well as insights into what resources would be helpful for providers and their patients. Feedback from this survey was used in the development of a website (*Connecting2Health* website) where providers and patients can go to seek educational content and access community resources for cardiometabolic disease management and prevention within the state of Alabama.

We anticipate that providers who engage in these educational interventions will attain improved knowledge and skills in the effective use of risk assessment tools and care guidelines. Webinars and training modules are recorded, archived, and made available online as enduring continuing education materials.

##### Data Transparency

To promote data transparency, practice facilitators work with each practice to extract clinical quality measures. The practice facilitators are well-versed in the clinical aspects of cardiovascular care, as well as the technical aspects of an EHR and data flow. They work with each practice independently to establish the best ways to extract the necessary clinical data without creating a new burden to providers or staff.

The ALCC contracted with the Consortium for Southeast Healthcare Quality (COSEHQ) to produce practice-level dashboards using data extracted from each practice’s EHR, which allows clinics to review their progress toward improving BP control and tobacco use screening and cessation intervention throughout the intervention period. Practice facilitators review the practice dashboards with practice staff as an additional method of tracking progress across the intervention period.

#### Clinical Outcomes

Outcomes of the study were selected based on their associations with patient-level benefit (ie, cardiovascular risk reduction) and avoiding undue burden for practices or providers to measure (Table 2). BP-related outcomes included documentation of hypertension in the EHR when a patient has 2 or more readings with a systolic BP≥130 mm Hg or a diastolic BP≥80 mm Hg, which aligns with the 2017 American College of Cardiology and American Heart Association BP guideline recommendation for hypertension diagnosis [27]. Given the high cardiovascular risk associated with stage II hypertension (systolic BP≥140 mm Hg or diastolic BP≥90 mm Hg) and the prevalence of stage II hypertension in Alabama, the main outcome for intervention efficacy is BP control among adults with hypertension to <140/90 mm Hg. This is also the BP target used in the Centers for Medicare & Medicaid Services (CMS) 165 and National Quality Forum (NQF) 0018 clinical quality measure. Tobacco use outcomes were available through the EHR and included tobacco use screening and cessation intervention. The combined measure of tobacco use screening and cessation intervention aligns with the CMS 138 and NQF 0028 clinical quality measures.

**Table 2:** Heart Health Improvement Project primary blood pressure and smoking cessation clinical outcomes.

Outcome	Description	Practice target
Hypertension control	Number of patients between 18 and 85 years of age diagnosed with hypertension, and a BP<140/90 mm Hg (consistent with controlled stage II hypertension)	≥70% of patients diagnosed with hypertension who have controlled stage II hypertension (BP<140/90 mm Hg)
Tobacco use screening	All patients ≥18 years of age have smoking status updated within past 2 years	≥75% of patients have smoking status documented per practice
		Rates of current smokers
		Rates of former smokers who relapse
		Rates of former and never smokers
		Rates of secondary smoking exposure
Tobacco use counseling	Use of 4 Rs^a^ to improve rates of smoking cessation	EHR^b^ documentation of smoking cessation counseling, referral to AL Quitline, medication documentation (including over-the-counter medication)
Tobacco use screening and counseling	Percentage of patients aged 18 years and older who were screened for tobacco use one or more times within 24 months and who received cessation counseling intervention if identified as a tobacco user	5-10% increase from baseline

^a^4 Rs: relevance, risks, rewards, roadblocks.

^b^EHR: electronic health record.

#### Measurement

Data on BP and tobacco use are available quarterly from the practice EHRs and retrospective EHR extraction provides preintervention data for all practices. In addition to clinical parameters, we collect data on age, race, and gender, as well as insurance status as available, to assess the impact of our program for these specific subgroups.

#### Statistical Analysis

The primary model for fixed effects without adjustment for any covariates will have the following structure:

Y=β0+β1(time)+β2(HHIP exposure)+β3(time after HHIP)

For the primary dichotomous outcomes of hypertension control and documentation of smoking status, Y will represent the log-odds of the binary outcome. In the model “time” will be considered as “calendar time” measured in quarters from the earliest quarter of baseline data and *β*_1_ will be the estimate of the secular trend. “HHIP exposure” will similarly measure the cumulative number of quarters the practice was exposed to HHIP, ranging from 0 in the baseline period, to 4 after a year of HHIP and *β*_2_ will be the estimate of the quarterly change due to HHIP. “Time after HHIP” will similarly quantify the period of follow-up after the year of HHIP and *β*_3_ will be the estimate for any enduring effect of HHIP on rates after the intervention beyond what could be attributed to secular trends. A positive *β*_3_ would indicate that outcomes continued to increase faster among practices after the HHIP intervention than would have been expected and would suggest a durable effect, while an estimate of *β*_2_ that is not significantly different from 0 would be interpreted as practices returning to the change expected by secular trends. In the general framework, the effect due to HHIP will be considered statistically significant if the *P* value of the model coefficient is <.05.

Before analysis, we will consider smoothed scatter plots of outcomes over time and HHIP exposure; if any trends appear to be importantly nonlinear, we will consider these as categorical to most appropriately model the data. If this is the case, the overall test of the main hypothesis will be the significance of the coefficient for the full-year exposure to HHIP. The general model can be further adjusted for patient-level characteristics (eg, age, race, and gender) and practice-level characteristics (eg, urban vs rural, FQHC vs non-FQHC, Consolidated Framework for Intervention Research [CFIR] scores, and other implementation-related measures) and stratified analyses can be run using subsets of the data. Differential effects by subgroups (eg, race, gender, or practice types) will be tested using interaction terms.

#### Power and Sample Size Considerations

Statistical power was assessed using simulation methods based on the outcome of hypertension control. Briefly, data was simulated for 50 practices, with randomly generated practice-level baseline hypertension control rates of 50% with an SD of 4%. Secular trends were similarly generated for each practice with a mean yearly decrease of 1% with an SD of 0.05%. We assumed a mean effect due to HHIP of 2% per year (0.5% per quarter) assigned randomly with an SD of 1%. Simulations included a range of residual HHIP effects ranging from 0.5% to 1.5%. Simulated datasets began with creating data for 10,000 patients per practice using generating baseline status at random, then iterated forward with each practice’s assigned secular trends and intervention effects, followed by the addition of random perturbations to model the high variability of BP measurements leading to changes in control status at each visit. For multiple iterations, 50 practices were randomly selected, along with a reduced sample of patients equal to 5 times the target sample size to reflect the fact that most patients will not have a visit in a given quarter (eg, if we expect to have data for 200 patients per practice per quarter, the data had an average of 1000 patients per practice). Finally, for each iteration, a random sample was drawn to reflect the mean of 200 patients per quarter. The mixed models were run on each iteration and power was estimated as the proportion of iterations in which coefficients were significant at the *P*<.05 level. We expect there to be >500 per patient per quarter, which would reflect <50 patients per week at a practice with a single practitioner so these power estimates should be highly conservative. Data from the Physicians Foundation in 2018 found that the average doctor sees 20 patients per day, so we could indeed expect >400 patients with hypertension per quarter for each physician [28]. Under these assumptions, there was >95% power for the HHIP effect with as few as 100 patients per site, which further suggests that we will have adequate power for analyses of patient subgroups analyses. Allowing for 400 patients per site provided 100% power for the secular trends and HHIP effect as well as 90% power for the 1% yearly residual increase from HHIP during follow-up. For analyses restricted to subgroups of practices, including only 25 practices with 400 patients per practice left >90% power for the secular trend and HHIP effect, but reduced power to 64% for the residual effect of HHIP during follow-up. Finally, in simulations of comparisons between subgroups (eg, race or gender), 50 practices with 400 patients per quarter per practice provide >80% power to detect a difference in yearly rates of 1.5% between groups (2% vs 3.5%) using a statistical interaction indicating that we will have adequate power for subgroup analyses. The outcomes related to smoking (principally documentation of smoking status) can be expected to have higher power based on higher baseline rates and greater control of the physicians (leading to lower variability in patient-level outcomes).

#### Evaluation of Implementation

The implementation outcomes evaluation will be guided by Proctor’s Framework for Implementation Outcomes [29], and we will assess implementation using the CFIR [21,30]. Our proposed evaluation of the implementation of the HHIP will be guided by the tenets of realist evaluation. The realist evaluation approach recognizes that there are many interwoven factors operating at different levels within a setting, making this evaluation method a better approach to complex interventions than a more traditional, noncontextual cause and effect evaluation approach [31].

Realist evaluation investigates the “context-mechanisms-outcomes pattern configuration.” Realist evaluation does not just consider outcomes to determine if an intervention is successful but analyzes the outcomes to discover if the speculated mechanism or context theories are confirmed. The overarching HHIP evaluation questions are: What works, for whom, in what way, to what extent, in what contexts, and how? To better understand context-specific mechanisms that may influence the successful implementation of HHIP, we will draw on a menu of constructs from the CFIR across 5 domains [21,30]. These domains include the (1) intervention characteristics, or aspects of the intervention that may have a positive or negative impact on implementation success, (2) outer setting, which includes the external forces that may influence successful implementation, (3) inner setting, which includes the organizational characteristics of the implementing organization, (4) characteristics of the individuals, including the knowledge, attitudes, beliefs, and personal attributes of those implementing, and (5) process of implementation, which refers to the stages of implementation and participation of key influencers in each of those stages.

Of the 39 constructs described in the CFIR, our evaluation will focus on the 23 constructs that are most relevant to this project (Table 3).

**Table 3:** Consolidated Framework for Implementation Research (CFIR) domains and constructs to be evaluated.

Intervention	Outer setting	Inner setting	Characteristics of individuals	Process
Complexity	External policies	Implementation Workflow Compatibility Learning climate Resistance to change	Knowledge and beliefs about the intervention	Planning
Relative advantage	Peer pressure	Leadership support	Self-efficacy	Reflecting and evaluating
Strength of adaptability	Patient needs Cosmopolitanism	Resources	Individuals to deliver the intervention Identification with the organization Individual stage of change	Champions Opinion leaders External change agents

Before implementation at each clinic, we administer the Organizational Readiness for Implementing Change (ORIC) survey, a psychometrically-validated instrument developed by Shea and colleagues [18], based on the constructs of Weiner’s Organizational Theory for Implementation Effectiveness [32]. At baseline and again at the conclusion of the HHIP, the evaluation team will survey the participating chief medical officer, a senior practice leader, or the practice champion of each clinic using the 14-item Change Process Capability Questionnaire (CPCQ) to assess a practice’s capacity to improve care using QI strategies [19].

Drawing on Proctor’s Conceptual Framework for Implementation Outcomes, we will evaluate the implementation of the HHIP. Proctor et al [29] describe 8 implementation outcomes, including acceptability, appropriateness, feasibility, costs, adoption, fidelity, penetration, and sustainability. We will address the acceptability, appropriateness, feasibility, and sustainability of the HHIP with validated questionnaires [33,34]. Fidelity and penetration will be assessed with administrative data collected as part of delivering the intervention (eg, number and duration of contacts with clinic). In addition, we will use semistructured interviews, approximately 30 minutes in duration and conducted through Zoom (Zoom Communications, Inc), with clinic staff members and providers from 8 to 10 clinics. Guided by the CFIR, the interviews will focus on the context for implementing the HHIP. Table 4 describes the outcomes that we will measure and our method or measurement.

**Table 4:** Implementation outcomes and methods of measurement.

Outcome	Definition	Measure
Acceptability	Perception that a given innovation is agreeable, palatable, or satisfactory	Acceptability of Intervention Measure (AIM): 4-item validated instrument
Appropriateness	The perceived fit, relevance, or compatibility of the evidence-based practice for a given practice setting and that it well addresses a particular problem	Intervention Appropriateness Measure (IAM): 4-item validated instrument
Feasibility	The extent to which an innovation can be successfully used given the setting	Feasibility of Intervention Measure (FIM): 4-item validated instrument
Adoption	The intention to employ an innovation of evidence-based practice	Measured at the provider level, the number of clinicians who intend to employ the innovation
Fidelity	The degree to which an intervention was implemented as it was prescribed	Measured at the provider level, adherence to the protocol including dosage and delivery
Penetration	The integration of a practice within a service setting	The number of eligible providers and staff who use an innovation divided by the number of providers and staff eligible to do so
Sustainability	The extent to which a newly implemented innovation becomes maintained or institutionalized in a service setting	Clinical Sustainment Assessment Tool (CSAT): 47 items across 7 domains

### Ethical Considerations

The study was approved by the Institutional Review Board at UAB (300006358 and 300007230). Each clinic entered into a business associate agreement with COSEHQ.

## Results

### HHIP Practice Recruitment

**Figure 3:**
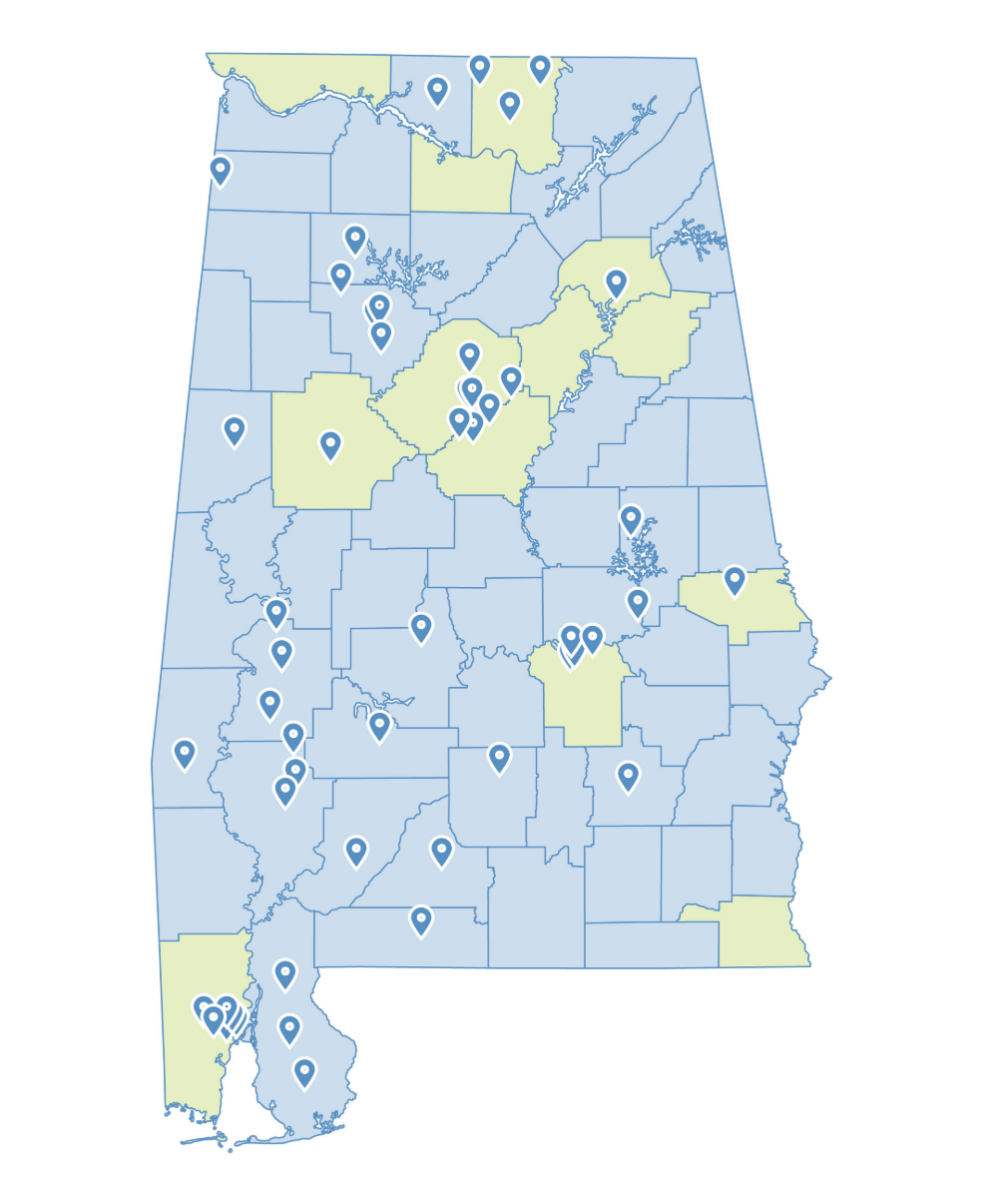
Location of primary care practices participating in the Heart Health Improvement Project. Pins indicate practice locations. Counties shaded in blue are classified as rural by the Alabama Rural Health Association.

Practice recruitment took place between April 2021 and October 2022. We contacted 417 primary care practices in Alabama (Figure 2). Of those, 51 practices declined to participate, and an additional 308 practices did not respond to our outreach. We excluded 7 practices that were in the process of having their eligibility determined at the time we reached our target enrollment. We enrolled 51 practices into the HHIP, including 28 FQHCs or look-alikes. Practices were geographically distributed across the state (Figure 3).

Of the 51 enrolled practices, 47 implemented the HHIP. Clinics that did not implement the HHIP included those that closed, lost a provider, or did not have sufficient staff time to dedicate to the intervention.

### HHIP Practice Characteristics

Among HHIP practices that completed baseline practice characteristics, 24% (11/45) were solo practices, while 62% (28/45) had 1 to 5 clinicians, and 13% (6/45) had 6 or more clinicians (Table 5). The median number of patient visits per year was 5819 (IQR 3707.3-8630.5). Practices had been in operation for a mean of 19.2 (range 1-50) years. Half (23/46, 50%) were certified as Patient-Centered Medical Homes. Overall, 72% (33/46) of practices share patient information and 78% (36/46) have a designated quality reporter. Overall, 76% (34/45) were reporting CMS 165 for control of high BP, while 44% (20/25) were reporting CMS 138 for tobacco use screening and cessation intervention.

**Table 5:** Heart Health Improvement Project practice characteristics.

Characteristics				Summary statistics	
**Practice characteristics**					
	**Practice size, n/N (%)**				
		Solo practice	11/45 (24)		
		2-5 clinicians	28/45 (62)		
		≥6 clinicians	6/45 (13)		
	**Practice ownership—FQHC^a^ or look-alike, n/N (%)**			28/47 (60)	
	**Single specialty, n/N (%)**			24/42 (57)	
	**Years of operation, mean (SD; range)**			19.2 (13.0; 1-50)	
	**Part of a network, n/N (%)**			26/46 (57)	
	**Number of visits per year, median (IQR)**			5819 (3707.3-8630.5)	
	**NCQA^b^ certified PCMH^c^, n/N (%)**			23/46 (50)	
	**Share patient health information, n/N (%)**			33/46 (72)	
	**Have a quality reporter for the practice, n/N (%)**			36/46 (78)	
	**Report CMS^d^ 165^e^, n/N (%)**			34/45 (76)	
	**Report CMS 138^f^, n/N (%)**			20/45 (44)	
**Baseline measures^g^, %**					
	Blood pressure control rate			49.6	
	Tobacco screening rate			81.8	
	Tobacco cessation intervention rate			71.4	
	Tobacco screening and cessation intervention rate			67.4	
**Baseline questionnaires, mean (SD)**					
	Change process capability questionnaire			11.6 (14.3)	
	Organizational readiness for implementing change				4.2 (0.84)

^a^FQHC: Federally Qualified Health Center.

^b^NCQA: National Committee for Quality Assurance.

^c^PCMH: Patient Centered Medical Home.

^d^CMS: Centers for Medicare & Medicaid Services.

^e^CMS 165: controlling high blood pressure.

^f^CMS 138: tobacco use screening and cessation intervention.

^g^Baseline measures were calculated using data collected from January 1, 2021, to the start of the intervention period. Data on blood pressure control are from 46 practices and data on tobacco screening and cessation intervention are from 44 clinics.

The results shown above do not encompass all currently enrolled practices, only those who have completed the baseline practice characteristics survey measures.

Baseline measures of BP control and tobacco use screening and cessation intervention were calculated using data collected from January 1, 2021, to the start of the intervention period. At baseline, the mean BP control rate was 49.6%. The mean tobacco use screening rate was 81.8% and the tobacco use cessation intervention rate was 71.4%; the rate of tobacco screening and cessation intervention was 67.4%. The mean CPCQ score was 11.6 (SD 14.3) and the median ORIC score was 4.2 (SD 0.84).

## Discussion

### Expected Findings

The ALCC represents a unique partnership of clinical, public health, and academic institutions that have come together to support primary care practices to reduce cardiovascular risk and disparities by race or ethnicity and rurality that persist in the state. The ALCC’s HHIP is a QI project that aims to improve the diagnosis and control of hypertension, documentation of tobacco use, and rate of tobacco cessation intervention in 47 primary care practices. We hypothesize that rates of BP control among adults with hypertension and tobacco use screening and cessation intervention will improve as a result of the HHIP, beyond what would be expected by secular trends.

Strengths of the HHIP include the multidisciplinary partnerships and multipronged approach to QI. Using evidence-based strategies, such as practice facilitation, on-site and e-learning, and data transparency, the HHIP intervention is designed to increase practices’ internal QI capacity and promote sustainability. The practice facilitation intervention was designed using the key driver model for care implementation and is delivered by trained facilitators. In addition, there is a robust plan to evaluate implementation outcomes. The HHIP clinics include FQHCs and look-alikes, with a number of them in rural areas. Including these clinics in the HHIP is essential for reducing disparities in the management of cardiovascular risk factors.

Limitations of the HHIP included delays in recruiting clinics, in part due to the COVID-19 pandemic, which forced a change in design from a true randomized stepped wedge to a pre-post trial design. Variability in staffing across clinics also means that capacity for QI initiatives will vary, which has implications for intervention implementation as well as sustainability. During the HHIP, clinics experienced staffing turnover, including provider changes or retirements and changes in practice champions. Finally, availability of data from the EHRs is also variable since clinics have different EHRs, and even when the EHR is the same, clinics may have purchased limited packages.

### Conclusions

If successful, the ALCC and HHIP may improve the implementation of evidence-based guidelines and, subsequently, cardiovascular health and health equity in the state of Alabama. The lessons learned can inform future primary care initiatives to improve cardiovascular health in the state of Alabama, as well as other state-based initiatives.

## Appendix

Multimedia Appendix 1 Key Drivers Implementation Scale.

Multimedia Appendix 2 Peer review report from the AHRQ Special Emphasis Panel - Agency for Healthcare Research and Quality (USA).

## Abbreviations

ALCC: Alabama Cardiovascular Cooperative
APHCA: Alabama Primary Health Care Association
BP: blood pressure
CMS: Centers for Medicare & Medicaid Services
CFIR: Consolidated Framework for Intervention Research
COSEHQ: Consortium for Southeast Healthcare Quality
CPCQ: Change Process Capability Questionnaire
CVD: cardiovascular disease
EHR: electronic health record
FQHC: Federally Qualified Health Centers
HHIP: Heart Health Improvement Project
NQF: National Quality Forum
ORIC: Organizational Readiness for Implementing Change
QI: quality improvement
UAB: University of Alabama at Birmingham

## Data Availability

The datasets generated during and/or analyzed during this study are available from the corresponding author on reasonable request.
