# Supplementary material for: Improving Blood Pressure Control and Tobacco Use Cessation Intervention In Primary Care: Protocol for the Alabama Cardiovascular Cooperative Heart Health Improvement Project
Source: JMIR Res Protoc. 2024 Dec 20;13:e63685. doi: 10.2196/63685 (PMC11699498; doi:10.2196/63685)
Supplement: Multimedia Appendix 1 [file resprot_v13i1e63685_app1.docx]

**Multimedia Appendix 1.** Key Drivers Implementation Scale.

| **KDIS Item** | **Construct(s) Assessed** | **Response Options** |
| --- | --- | --- |
| Clinical Information System | Extent to which a practice uses data from their electronic health record or registry for population health management | 0 = Practice currently does not review practice population data, such as a report that shows how many patients have hypertension or how many have it under control. |
|  |  | 1 = Practice has access to reliable data on their patients with hypertension (for example, all patients with hypertension and their average BP, or the % of hypertension patients that have BP <140/90) |
|  |  | 2 = The practice trusts their BP data / reports enough to consider implementing change activities |
|  |  | 3 = The practice accesses and reviews BP data monthly and discusses how to make changes to improve processes to optimize BP control |
| Optimized Team Care | Extent to which practice team members share workloads for patient care and quality improvement activities | 0 = No QI activities related to hypertension currently |
|  |  | 1 = Occasional meetings or discussions regarding QI for hypertension but no practice-wide understanding of QI |
|  |  | 2 = A QI team communicates regularly (through meetings, huddles, emails, memos, etc.) to plan tests and discuss results of hypertension QI. QI team can describe project focus and measures. |
|  |  | 3 = A QI team is planning and discussing multiple tests simultaneously to improve hypertension control, and communicates findings to each other. QI progress is communicated to entire office staff. Most staff can describe QI focus and measures. |
| Standardized Care Processes | Extent to which a practice uses evidence-based or informed protocols to standardize treatment | 0 = The practice currently has no activity on following evidence-based protocols for hypertension. |
|  |  | 1 = The practice has identified one or more evidence-base or best practice protocol(s) for hypertension, and has begun the process of customizing one or more protocols for their own practice to guide care for their patients with high blood pressure. |
|  |  | 2 = The practice has established a workflow to support implementing at least one hypertension protocol and it has been tested on at least a few patients. |
|  |  | 3 = The practice has implemented an evidence-based protocol for hypertension, but it is not yet being used with all patients. |
|  |  | 4 = The practice routinely fully implements and follows at least one evidence-based protocol for hypertension. |
| Self-Management Support | Extent to which a practice uses resources to enable patients to self-manage their health condition | 0 = Practice currently has no activity on self-management support for patients with hypertension |
|  |  | 1 = Practice staff understands the difference between patient education and self-management support |
|  |  | 2 = Practice identifies hypertension related self-management support resources and incorporates the use of the resources into their workflow |
|  |  | 3 = Practice develops tracking systems to monitor use of hypertension related self-management support resources. |
|  |  | 4 = The care team 1) collaborates with patients to set hypertension related self-management goals, 2) documents the goals, and 3) reviews previous goals at every visit. |
|  |  | 5 = Care team assess patients’ confidence level related to managing their hypertension. |
| Leadership | Extent to which a practice has leadership support for quality improvement activities | 0 = No management or leadership support for QI work in hypertension currently exists.\| |
|  |  | 1 = A single manager or physician champion is involved but no organized QI structure for hypertension exists. “Try and see approach” is the norm for QI activities related to hypertension. |
|  |  | 2 = The practice has a leader who supports hypertension QI activities and there are some tasks that are assigned to staff members. |
|  |  | 3 = QI work for hypertension is integrated into daily routines and there are certain staff who are assigned QI activities. |

BP, blood pressure; KDIS, Key Drivers Implementation Scale; QI, quality improvement. Adapted from Stover et al, 2022.[26]
