# Supplementary material for: Improving Blood Pressure Control and Tobacco Use Cessation Intervention In Primary Care: Protocol for the Alabama Cardiovascular Cooperative Heart Health Improvement Project
Source: JMIR Res Protoc. 2024 Dec 20;13:e63685. doi: 10.2196/63685 (PMC11699498; doi:10.2196/63685)
Supplement: Multimedia Appendix 2 [file resprot_v13i1e63685_app2.pdf]

1U18HS027946-01 Cherrington, Andrea

RESUME AND SUMMARY OF DISCUSSION: The following application is submitted by Dr. Andrea Cherrington, Professor of Medicine at the University of Alabama at Birmingham School of Medicine. The application responds to RFA-HS-20-002 and titled "The Alabama Cardiovascular Cooperative: Supporting Cardiovascular Risk Reduction in Primary Care." The application establishes the Alabama Cardiovascular Cooperative that features sustainable partnerships and stakeholder engagement to involve primary care clinics in cardiovascular risk reduction while implementing and evaluating the impact of a heart health improvement program in 60 primary care clinics. The reviewers were highly enthusiastic about the study and considered it very significant because Alabama has a high cardiovascular disease (CVD) burden leading to death, hospitalizations and emergency department visits so if successful, findings have the potential to improve CVD burden and produce information that can be implemented in other states interested in improving state capacity to provide external quality improvement (QI) support to primary care practices. The panel noted the investigator team as strong with complimentary expertise with a principal investigator that is clinically trained in health services research and diabetes. Other strengths identified by the panel include a study highly responsive to the RFA, a cooperative that will reduce health disparities, strong letters of support submitted by their partners, a highly detailed approach to establishing the cooperative, provisions put in place to bolster telehealth sites in the event that COVID-19 impacts the study, and an excellent environment. The only minor concern expressed by the reviewers centered around Dr. Cherrington's low effort on the project, which is currently 15 percent. Overall, the panel agreed the study is highly significant with many strengths with the study's findings likely to exert a high impact on improving cardiovascular risk reduction and implementation of a heart health improvement program in primary care clinics in the state of Alabama.

DESCRIPTION (provided by applicant):
In the United States (U.S.), the last three decades have seen vast improvements in cardiovascular disease (CVD) risk and mortality, however, these gains are not consistent across all regions of the country. Inequities in cardiovascular outcomes remain, and in some cases have widened, across racial, socio-economic, and geographic lines. In the Southeastern U.S., rates of stroke, myocardial infarction (MI), diabetes and obesity are among the highest in the nation. According to data from millionhearts.org, Alabama has the highest rate of CVD events (death, hospitalizations, ED visits) among all 50 states. Although evidence-based guidelines for screening and treatment of cardiovascular risk factors exist, implementation is often suboptimal. Many factors contribute to the research-to-practice gap, including educational deficiencies, time constraints for providers, lack of feedback mechanisms and decision support tools, as well as cultural factors and organizational climate. There is potential for real impact in CVD health if primary care providers and patients are provided with tools to address cardiovascular health (i.e., assess and manage cardiovascular risks), particularly in the rural south. With this application, we propose the creation of the Alabama Cardiovascular Cooperative to promote coordination of statewide efforts to improve cardiovascular risk and reduce disparities. Partners include 5 organizations with long-standing commitments to improving health outcomes in Alabama, broad statewide reach, and a shared belief that a coordinated effort to support primary care-based initiatives to improve CVD health in Alabama could prove transformative; these entities include the Alabama Department of Public Health (ADPH), the Alabama Quality Assurance Foundation (AQAF), the Alabama Primary Health Care Association (APHCA), Auburn University School of Pharmacy, and the University of Alabama at Birmingham School of Medicine (UAB). We will engage primary care practices from across the state in a sustainable, CVD-focused "community of practice." We will implement a Heart Health Improvement project in 60 primary care clinics with suboptimal rates of blood pressure (BP) control and screening for smoking status using a 3-prongued approach

comprising Practice Facilitation & Technical Assistance, Online & eLearning, and Improvement through Data Transparency. We will use a Type I hybrid design, to simultaneously test the effects of the intervention on BP and smoking-related outcomes while also gathering information on implementation. The outcomes evaluation will be guided by the Proctor's Framework for Implementation Outcomes, and we will assess implementation using the Consolidated Framework for Implementation Research. Through these coordinated efforts, our overarching goal is to propel Alabama out of the bottom quartile for cardiovascular outcomes and reduce rural/urban and racial disparities.

PUBLIC HEALTH RELEVANCE
Alabama has the highest rate of cardiovascular (CVD) events (death, hospitalizations, ED visits) among all 50 states. We propose the creation of the Alabama Cardiovascular Cooperative, a sustainable, community- academic partnership to promote coordination of statewide efforts to improve cardiovascular risk and reduce disparities.

CRITIQUE NOTE: The sections that follow are the essentially unedited, verbatim comments of the individual committee members assigned to review this application. The attached commentaries may not necessarily reflect the position of the reviewers at the close of group discussion, nor the final majority opinion of the group. The above RESUME/SUMMARY OF DISCUSSION represents the evaluation of the application by the entire committee.

CRITIQUE 1

Significance: 1
Investigator(s): 1
Innovation: 2
Approach: 2
Environment: 1

Overall Impact

This application is designed to establish the Alabama Cardiovascular Cooperative, engage primary care clinics in a cardiovascular risk reduction network and implement & evaluate the impact of a Heart health Improvement program in 60 primary care clinics in Alabama. Significance includes focus on reducing burden of CVD is a state with an exceedingly high CVD event rate and prevalence of potentially modifiable CVD risk factors. Strengths include the assembled multidisciplinary team, proposed approaches for establishing the Cardiovascular Cooperative (ACC), plans for engagement of non-traditional sources of primary care services, approach to HHIP & implementation Logic Model proposed plans for evaluation.

1. Significance

Strengths

- The application is very responsive to the RFAs goal of creating primary care QI support capacity in Alabama, a state with an exceedingly high burden of CVD
- Information included addresses (in detail) the burden of CVD in Alabama, a state with highest rates of CVD events (death, hospitalizations, ED visits) among all 50 states.
- Creation of the Alabama Cardiovascular Cooperative as presented is designed to improve cardiovascular risk reduction and reduce disparities
- Partners identified (with strong letters of support) include 5 organizations with long-standing commitments to improving health outcomes in Alabama (Alabama Dept of Public Health, Alabama Quality Assurance Foundation (AQAF),Alabama Primary healthcare Association, Auburn University School of Pharmacy, & UAB School of Medicine.
- Plans to engage primary care practices across the state in a sustainable CVD-focused "community of Practice "
- Plans to implement Heart Health Improvement Project in 60 primary care clinics.
- Focus on BP control and smoking , potentially modifiable major risk factors for CVD
- Use of Proctor's Framework to guide Implementation outcomes evaluation & assessment of implementation using the Consolidated Framework for Implementation Research.

Weaknesses
- None noted

2. Investigator(s)

Strengths
- The assembled multidisciplinary, multisite team has the experience and expertise to conduct this project as proposed and is viewed as a major strength of this application
- PI Andrea Cherrington, MD,MPH, is a clinician trained in health services research as well as community-based participatory research methods. Her research has focused on developing, implementing & evaluating community-based interventions for chronic disease management with emphasis on underserved communities. Currently she serves as Director, Intervention & Translation Core, UAB's NIH-funded Diabetes research center , directs the diabetes clinic in Cooper Green health System and is recently named Director of Clinical & Population Science Program at UAB/Medicine. Her publications are relevant to the content and methods proposed in this application.
- Co-PI Elizabeth Jackson, MD, MPH, is Director of Cardiovascular Outcomes & Effectiveness Research Program at UAB. She is also chair-elect of AHA's Social Determinants of Health Committee. Currently (NCX) she is funded by NIH for an RCT internet-mediated walking program for individuals with PAD and serves as co-I, UAB's Cardiovascular Collaboration, and co-I on UAB's Diabetes Research Center.
- Thomas Creger, PhD, MPH, is Evaluation Co-Director, Research & Informatics Training center. A public health scientist with research emphasis on program implementation, evaluation and program monitoring and experience/expertise in multi-level prevention interventions, he is well-qualified to lead the proposed Evaluation Core in this project.
- Richard Hansen, PhD, Dean & Endowed Professor, School of Pharmacy, is a health services researcher with emphasis on pharmaceutical outcome research (comparative effectiveness & safety of drug products). He has the experience/expertise and familiarity with practice sites & clinical infrastructure relevant to medicate usage in Alabama central to his role in this project. He will co-direct the Engagement Core (with PI Cherrington).
- Sharon Parker, BSN, RN, Chief Quality Officer, Alabama Primary Health Care Association, has substantial experience in nursing/healthcare with emphasis on management & operational

capacity. She is well-prepared & positioned to serve as co-lead of the Implementation Core of this project.
- Lee Pearce, MSW,MSHA, Vice-President, Alabama Quality Assurance Foundation. Her experience and expertise make her well-suited to serve as co-lead on the Implementation Core of the proposed project.
- Members of the assembled team have and continue to collaborate on projects relevant to the proposed application.
- Effort for the key personnel appear appropriate to roles and for duration of the project.

Weaknesses
- None noted

3. Innovation

Strengths
- Plans for developing state-level capacity to deliver QI primary care practice support in Alabama are viewed as moderately innovative and much needed in the state. Of note > 90 % of practices in Alabama are underserved or rural (as indicated by CMS). Currently there is one practice-based contract for QI efforts in Alabama receiving federal funding.
- Partners in this application (APHCA, AQAF, ADPH) have and continue to be engaged in QI efforts in the state. ADPH , for example, has played a role in identifying and prioritizing QI goals at the state level with Practice Management/Quality Improvement unit working with all ADPH programs including the Cardiovascular Disease & Diabetes programs and are currently working to improve hypertension control (a major goal of this application) via improving access to BP self- monitoring sites.
- Opportunities for alignment with public health initiatives & community-based resources in Alabama are identified.
- Approaches to dissemination & implementation allowing for adaptation at the practice level are included

Weaknesses
- None noted

4. Approach

Overall Approach

Strengths
- Plans identified for launching the project including establishing a Cooperative , building the network engaging with primary practices are well-detailed and appear appropriate to the aims and feasible within the projected timeline.
- As presented, the proposed project is likely to achieve goal of expanding capacity to provide QI support to primary care practices within Alabama.
- Moderate- to high likelihood of D & I of PCOR findings to primary care practices.

Weaknesses
- None noted

Approach to Establishing the Cooperative

Strengths

- Convening of partners with statewide reach and commitment to reduction of CV risk and disparities
- Use of strategies from the framework for Community- Academic Partnerships – designed to improve community outcomes through equal partnerships among academic, community stakeholders in all phases of project and enhance/promote sustainability
- The proposed Cooperative Organizational structure with partnerships among UAB ,AQAF, APHCA, Auburn University & ADPH has commitments with LOS from the lead of each institution.
- An Advisory Board that includes representation from key stakeholders from multiple sectors across Alabama with plans to provide guidance via quarterly meetings and PRN communication as needed.
- Consortium for Southeast Healthcare Quality (COSEHQ), a non-profit founded in 1994 and designed to address the burden of CVD in Southeast, will provide expertise and consultation.
- Taken together, information provided indicates a viable plan for bringing together diverse organizations/groups as well as resources to form a Cooperative that also includes a clear and strong governance structure with robust plans for project management , engagement & decision-making

Weaknesses

- None Noted

Approach to Establishing a Network of Primary Care Practices

Strengths

- Cooperative's plans to engage primary care practices as well as non-traditional providers of primary care in the Cardiovascular Risk Reduction Network (CRRN).
- Community of Practice (CoP) , forum for communication on best practices
- Plans to engage Deep South network, currently supported by UAB administration, that includes ~ 1300 health care providers (~60% are PCPs)
- Engaging non-traditional sources of primary care services: HealthWatch- a worksite wellness program that uses community pharmacies as a screening site

Weaknesses:

- None Noted

Approach to Heart Health Improvement Project

Strengths

- Cooperative's plans to use existing communication channels for recruitment of primary care practices in Heart Health Improvement Project (HHIP).
- Plans to identify and engage a Practice Champion as part of enrollment processes
- Prior related initiatives demonstrate feasibility: prior work has less than 5% attrition
- Plans to require at least 50% of clinics are FQHCs and at least 50% are in rural areas
- Well-defined Logic Model for HHIP Implementation- flows from implementation strategies to mechanisms and outcomes
- Application/integration of Chronic Care Model and core components to optimize care of patients with chronic conditions (CVD).
- Plans for technical support & practice facilitation

Weaknesses:
- None Noted

Approach to Evaluation

Strengths
- Evaluation plan is well defined and detailed and includes an examination of the establishment of statewide Cooperative, and its processes as well as products.
- Plans for evaluation of the Cardiovascular Risk Reduction Network including tracking of number of letters of agreement signed compared to number of clinics approached.
- Evaluation questions proposed & plans for collecting /evaluating internal & external information and use of mixed methods (quantitative & qualitative approaches) to evaluation
- Effectiveness- implementation hybrid design planned to evaluate HHIP (seeking to test the effects of a clinical intervention on outcomes while also gathering information on implementation
- Use of Consolidate Framework for Implementation Science to understand the contextual-specific mechanisms that have potential to influence implementation of HHIP
- Sustainability evaluation

Weaknesses
- None Noted

Approach to Dissemination

Strengths
- Multifaceted dissemination plan that spans all aspects of the proposed project including Cooperative activities, Primary Care Cardiovascular Risk reduction network and the HHIP.
- Applicants have clearly indicated a commitment to cooperate with AHRQ and its contractors on dissemination efforts.

Weaknesses:
- None noted

5. Environment

Strengths
- The environments at UAB, Auburn and other sites involved in this application are viewed as major strengths of this application with resources and supports particularly well-suited for addressing the major aims as proposed.

Weaknesses
- None noted

ADDITIONAL REVIEW CRITERIA

How well does the proposed research plan align with AHRQ’s mission and research priorities?

Strengths
- Superb alignment with mission and goals of AHRQ

Weaknesses
- None noted

Protections for Human Subjects

Strengths:
- Acceptable as presented; exempt # 2 criterion

Weaknesses
- None Noted

The requirement for the single IRB review (45 CFR 46.114)
For non-exempt human subjects research projects:
The project constitutes cooperative research that is subject to the requirement for single IRB review: NO
If the answer is yes, Is a single IRB plan included in the application:
Yes No
If the answer is No, a single IRB plan must be submitted for AHRQ review and approved before an award can be made.

The HHS regulations on the protection of human subjects in research at 45 CFR 46.114 (b) requires that any institution located in the United States that is engaged in cooperative research as defined under 45 CFR 46.114 (a) must rely upon approval by a single IRB for that portion of the research that is conducted in the United States. The reviewing IRB will be identified by the Federal department or agency supporting or conducting the research or proposed by the lead institution subject to the acceptance of the Federal department or agency supporting the research. Refer to the Revised Common Rule at https://www.ecfr.gov/cgi-bin/retrieveECFR?gp=&SID=83cd09e1c0f5c6937cd9d7513160fc3f&pitd=20180719&n=pt45.1.46&r=PART&ty=HTML, and the OHRP's guide on the single IRB requirement at https://www.hhs.gov/ohrp/regulations-and-policy/single-irb-requirement/index.html. AHRQ Guide Notice at https://grants.nih.gov/grants/guide/notice-files/NOT-HS-20-005.html.

Posting Clinical Trial Informed Consent Form

Does this project is/involves clinical trial as defined under 45. CFR.46. 102(b): NO

If this project is/involves clinical trial as defined under 45 CFR 46.102(b), please be reminded that for clinical trials conducted by or supported by a Federal department or agency, Section 46.116(h) of the Revised Common Rule requires the posting of an IRB-approved consent form on a public federal website designated for posting such consent forms. To fulfill this requirement, recipients conducting AHRQ-funded clinical trials must submit one IRB-approved version of a consent form that has been used to enroll participants to either ClinicalTrials.gov, or the Regulations.gov website. Refer to AHRQ Guide Notice at https://grants.nih.gov/grants/guide/notice-files/NOT-HS-19-023.html for more information.
Clinical trial means a research study in which one or more human subjects are prospectively assigned to one or more interventions (which may include placebo or other control) to evaluate the effects of the interventions on biomedical or behavioral health-related outcomes (45 CFR 46.102(b)).
(if not please comment on Informed consent): N/A

Degree of Responsiveness: Highly responsive to AHRQ mission & goals and to this FOA

Budget and Period of Support

Strengths
- Approve as submitted

Weaknesses
- None Noted

Inclusion of Priority Populations: Acceptable as presented
Weaknesses

Authentication of Key Resources used: N/A

CRITIQUE 2

Significance: 1
Investigator(s): 1
Innovation: 3
Approach: 2
Environment: 1

Overall Impact

Strengths
- This is a very strong application from an established team of investigators from Alabama, the state with the highest CVD burden in the country. Proposed Cooperative builds on the existing partnerships between UAB, Auburn University, Alabama Quality Assurance Foundation and Alabama Primary Health Care Association. In particular, partnership with the last two entities makes it likely to achieve the goal of expanding the capacity to provide external QI support to primary care practices, and is to continue the expanded capacity beyond the project period. From the previous projects, they demonstrate the ability to recruit and retain practices for implementation of interventions. Approach to Heart Health Improvement Project is very solid and thoughtful. Evaluation plans are detailed and sound. This project has a very high likelihood of success.

Weaknesses
- There are some minor weaknesses in BP intervention but these do not diminish the enthusiasm for this project.

1. Significance
Strengths

- Alabama has the highest rate of CVD events (death, hospitalizations, ED visits) among all 50 states.
- The proposal is highly responsive to the RFA's goal of creating and sustaining external primary care QI support capacity in Alabama.
- A clear description of the context of primary care delivery in Alabama, including the State's CVD burden is provided.
- This project has a high potential to produce information and tools useful to others that are interested in improving a State's capacity to provide external QI support to primary care practices and to disseminate and implement PCOR findings in primary care practice.

Weaknesses
- None Noted

2. Investigator(s)

Strengths
- Dr. Cherrington, Professor in DOPM and General Internal Medicine at UAB is superbly qualified to lead this program. She also serves as Director of the UAB Clinical and Population Health Sciences program as well as the Director of the Intervention and Translational Core within the UAB Diabetes Research Center. She lists 70 publications and has been PI/ coinvestigator in several federally funded projects. She led the Southeastern Collaboration to Improve Blood Pressure Control project, which enrolled 69 practices in a trial to improve blood pressure control through the use of Practice Facilitation and/or Peer Coaching.
- A multidisciplinary team of coinvestigators includes Drs. Jackson, Creger and Richman at UAB, Drs. Hansen and Hohmann at Auburn, Ms. Parker at Alabama Primary Health Care Association and Mr. Pearce at Alabama Quality Assurance Foundation.

Weaknesses
- None Noted

3. Innovation

Strengths
- The application is solid but not innovative. It will use existing partnerships and existing resources. This is not a weakness but a strength of the proposal.

Weaknesses
- None noted

4. Approach

Overall Approach

Strengths
- Given the existing partnerships and the experience of the investigative team, they are likely able to launch the project quickly and complete the project in the time frame.
- Partnership with Alabama Quality Assurance Foundation and Alabama Primary Health Care Association makes it likely to achieve the goal of expanding within the State the capacity to provide external QI support to primary care practices, and is to continue the expanded capacity beyond the project period.

- The proposed approach likely to result in dissemination and implementation of PCOR findings to primary care practices

Weaknesses
- None Noted

Approach to Establishing the Cooperative

Strengths
- This strong Cooperative will be led by UAB with Auburn University, Alabama Quality Assurance Foundation and Alabama Primary Health Care Association as participating institutions.
- The involvement of Alabama Department of Public Health ADPH augurs well for the success of the project.
- There are strong letters of support from the proposed members of the Cooperative and State organizations.
- Executive Committee will consist of the Organization and Program Leads. It includes all participating sites.
- Proposed members of the Cooperative have the necessary expertise and track record of implementing similar interventions in community practices.

Weaknesses
- None Noted

Approach to Establishing a Network of Primary Care Practices

Strengths
- They plan to recruit 60 clinics with suboptimal rates of HTN control and screening for smoking status networks associated with APHCA, AQAF, as well as those involved in their previous trials.
- They have a track record of recruiting PC clinics.
- They also propose recruiting non-traditional primary care sources such as wellness programs and rural pharmacies. This is a strength.

Weaknesses
- None Noted

Approach to Heart Health Improvement Project

Strengths
- They propose to recruit 60 primary care clinics with suboptimal rates of BP control and screening for smoking status.
- A 3-prongued approach comprising Practice Facilitation & Technical Assistance, Online & eLearning, and Improvement through Data Transparency is proposed.
- The intervention consists of trained practice facilitators working with 60 clinic’s staff to generate a list of practice level outcomes related to HTN risks that are currently suboptimal. PFs will interact with each practice once a month for one year and assist the practices as they access their electronic health records to obtain baseline assessment of frequency of screening, testing, management and referrals. An implementation plan will be tailored to each practice based on the evaluation data. This is feasible.

- The use of type I hybrid design, to simultaneously test the effects of the intervention on BP and smoking-related outcomes while also gathering information on implementation.
- The outcomes evaluation will be guided by the Proctor's Framework for Implementation Outcomes.
- They will assess implementation using the Consolidated Framework for Implementation Research.
- Recognition of the challenges of disparate EHR and a plan to address this with monetary support to the clinics is very thoughtful.

Weaknesses
- While the details of implementation are very thoughtfully described, the specific details of BP intervention are not well developed. Will patients be trained to accurately measure BP at home? Will office BP measurements be standardized? Will there be recommended antihypertensive agents to be adopted as first line?

Approach to Evaluation

Strengths
- There is a very strong evaluation plan that very well addresses measurement, data collection and management, and analytic plans adequate to answer proposed evaluation questions.
- They propose to use Consolidated Framework for Implementation Science describing internal and external contextual factors that could influence the effectiveness of the approach.
- Hypertension is defined as documentation in the EHR when a patient has 2 or more reading with a systolic BP of 130mmHg or greater or a diastolic BP of 80mmHg or greater. This is appropriate.
- Statistical methods are appropriate.
- Secular trends are considered in the plan.

Weaknesses
- The definition of HTN is based on two BP readings in office. Are these consecutive readings? Are they measured within a given time interval or any two BP readings?
- Will the practices be encouraged to re-measure BP in the clinic after 5 minutes of rest if the BP reading is high?

Approach to Dissemination

Strengths
- Dissemination plan is well written and feasible.

Weaknesses
- None Noted

5. Environment

Strengths
- UAB has an outstanding environment to carry out the proposed project.
- The applicant effectively leverages State and local expertise, infrastructure, and resources with partnerships with ADPH, Alabama Primary Health Care Association, Alabama Quality Assurance Foundation and Auburn University.

- The availability of Intelligent Care Platform, a flexible, real-time, health information
- System that integrates clinical and operational data from disparate systems is a real strength.

Weaknesses
- None Noted

ADDITIONAL REVIEW CRITERIA

How well does the proposed research plan align with AHRQ's mission and research priorities? N/A

Protections for Human Subjects: N/A

The requirement for the single IRB review (45 CFR 46.114): N/A
For non-exempt human subjects research projects:
The project constitutes cooperative research that is subject to the requirement for single IRB review:
YES NO
If the answer is yes, Is a single IRB plan included in the application:
Yes No
If the answer is No, a single IRB plan must be submitted for AHRQ review and approved before an award can be made.

The HHS regulations on the protection of human subjects in research at 45 CFR 46.114 (b) requires that any institution located in the United States that is engaged in cooperative research as defined under 45 CFR 46.114 (a) must rely upon approval by a single IRB for that portion of the research that is conducted in the United States. The reviewing IRB will be identified by the Federal department or agency supporting or conducting the research or proposed by the lead institution subject to the acceptance of the Federal department or agency supporting the research. Refer to the Revised Common Rule at https://www.ecfr.gov/cgi-bin/retrieveECFR?gp=&SID=83cd09e1c0f5c6937cd9d7513160fc3f&pitd=20180719&n=pt45.1.46&r=PART&ty=HTML, and the OHRP's guide on the single IRB requirement at https://www.hhs.gov/ohrp/regulations-and-policy/single-irb-requirement/index.html. AHRQ Guide Notice at https://grants.nih.gov/grants/guide/notice-files/NOT-HS-20-005.html.

Posting Clinical Trial Informed Consent Form: N/A

Does this project is/involves clinical trial as defined under 45. CFR.46. 102(b): YES NO

If this project is/involves clinical trial as defined under 45 CFR 46.102(b), please be reminded that for clinical trials conducted by or supported by a Federal department or agency, Section 46.116(h) of the Revised Common Rule requires the posting of an IRB-approved consent form on a public federal website designated for posting such consent forms. To fulfill this requirement, recipients conducting AHRQ-funded clinical trials must submit one IRB-approved version of a consent form that has been used to enroll participants to either ClinicalTrials.gov, or the Regulations.gov website. Refer to AHRQ Guide Notice at https://grants.nih.gov/grants/guide/notice-files/NOT-HS-19-023.html for more information.
Clinical trial means a research study in which one or more human subjects are prospectively assigned to one or more interventions (which may include placebo or other control) to evaluate the effects of the interventions on biomedical or behavioral health-related outcomes (45 CFR 46.102(b)).

(if not please comment on Informed consent): N/A

Degree of Responsiveness: N/A

Budget and Period of Support
Strengths
- The budget is very thoughtfully put together. Appropriate budgetary support is requested.

Weaknesses
- None Noted

Inclusion of Priority Populations: N/A

Authentication of Key Resources used: N/A

CRITIQUE 3

Significance: 1
Investigator(s): 4
Innovation: 3
Approach: 6
Environment: 2

Overall Impact

Strengths
- The PI and coPIs have a low amount of requested funding from the project to carry out such an ambitious project, but the PI has a track record of success of related diabetes research. Cherrington's publication record outstanding with 70 publications.
- COVID is mentioned as a possible limitation for enrollment, with funds earmarked for bolstering telehealth for sites that lack it.

Weaknesses
- None Noted

1. Significance

Strengths
- Alabama = #2 state re:CVD burden in the US
- Specifically targets rural practices with high burden of underserved in the US

Weaknesses

- None Noted

2. Investigator(s)

Strengths

- the PI has a track record of success in similar diabetes research
- Creger (Evaluation) has experience and 20% funding with proposal for the evaluation

Weaknesses

- The PI and coPIs have a low amount of requested funding from the project to carry out such an ambitious project,

3. Innovation

Strengths

- Alabama would greatly benefit from the establishment of a CVD cooperative.

Weaknesses

- None Noted

4. Approach

Overall Approach

Strengths

- the PI has a track record of success in diabetes research

Weaknesses

- The PI and coPIs have a low amount of requested funding from the project to carry out such an ambitious project

Approach to Establishing the Cooperative

Strengths

- PI has track record of success with implementation of related/similar diabetes initiatives

Weaknesses

- None Noted

Approach to Establishing a Network of Primary Care Practices

Strengths

- Proposal has high feasibility for success, as recruitment of 50/60 primary care practices is proposed; Track record of Past attrition rate of only 5%

Weaknesses

- None Noted

Approach to Heart Health Improvement Project

Strengths
- Established CV education/improvement tools for use are proposed for implementation and dissemination

Weaknesses
- None noted

Approach to Evaluation

Strengths
- Evaluation tools are sound and thorough as proposed, including examination of the establishment of statewide Cooperative, outcomes;the Cardiovascular Risk Reduction Network recruitment and outcomes

Weaknesses
- None Noted

Approach to Dissemination

Strengths
- Cherrington's publication record outstanding with 70 publications.

Weaknesses
- Cherrington's publication record is modest, with studies from 2016 or older.

5. Environment

Strengths
- None Noted

Weaknesses
- None Noted

ADDITIONAL REVIEW CRITERIA

How well does the proposed research plan align with AHRQ's mission and research priorities?

Strengths
- Well aligned with AHRQ goals

Weaknesses
- None Noted

Protections for Human Subjects: Acceptable

The requirement for the single IRB review (45 CFR 46.114)

For non-exempt human subjects research projects:
The project constitutes cooperative research that is subject to the requirement for single IRB review:
YES
If the answer is yes, Is a single IRB plan included in the application:
Yes
If the answer is No, a single IRB plan must be submitted for AHRQ review and approved before an award can be made.

The HHS regulations on the protection of human subjects in research at 45 CFR 46.114 (b) requires that any institution located in the United States that is engaged in cooperative research as defined under 45 CFR 46.114 (a) must rely upon approval by a single IRB for that portion of the research that is conducted in the United States. The reviewing IRB will be identified by the Federal department or agency supporting or conducting the research or proposed by the lead institution subject to the acceptance of the Federal department or agency supporting the research. Refer to the Revised Common Rule at https://www.ecfr.gov/cgi-bin/retrieveECFR?gp=&SID=83cd09e1c0f5c6937cd9d7513160fc3f&pitd=20180719&n=pt45.1.46&r=PART&ty=HTML, and the OHRP's guide on the single IRB requirement at https://www.hhs.gov/ohrp/regulations-and-policy/single-irb-requirement/index.html. AHRQ Guide Notice at https://grants.nih.gov/grants/guide/notice-files/NOT-HS-20-005.html.

Posting Clinical Trial Informed Consent Form

Does this project is/involves clinical trial as defined under 45. CFR.46. 102(b): YES

If this project is/involves clinical trial as defined under 45 CFR 46.102(b), please be reminded that for clinical trials conducted by or supported by a Federal department or agency, Section 46.116(h) of the Revised Common Rule requires the posting of an IRB-approved consent form on a public federal website designated for posting such consent forms. To fulfill this requirement, recipients conducting AHRQ-funded clinical trials must submit one IRB-approved version of a consent form that has been used to enroll participants to either ClinicalTrials.gov, or the Regulations.gov website. Refer to AHRQ Guide Notice at https://grants.nih.gov/grants/guide/notice-files/NOT-HS-19-023.html for more information.
Clinical trial means a research study in which one or more human subjects are prospectively assigned to one or more interventions (which may include placebo or other control) to evaluate the effects of the interventions on biomedical or behavioral health-related outcomes (45 CFR 46.102(b)).
(if not please comment on Informed consent): N/A

Degree of Responsiveness: Responsive

Budget and Period of Support: Reasonable

Inclusion of Priority Populations

Strengths
- Disadvantaged populations will be targeted per FOA

Weaknesses
- None Noted

Authentication of Key Resources used: NA

THE FOLLOWING SECTIONS WERE PREPARED BY THE SCIENTIFIC REVIEW OFFICER TO SUMMARIZE THE OUTCOME OF DISCUSSIONS OF THE REVIEW COMMITTEE, OR REVIEWERS' WRITTEN CRITIQUES, ON THE FOLLOWING ISSUES:

PROTECTION OF HUMAN SUBJECTS: ACCEPTABLE

INCLUSION OF WOMEN PLAN: ACCEPTABLE

INCLUSION OF MINORITIES PLAN: ACCEPTABLE

INCLUSION ACROSS THE LIFESPAN: ACCEPTABLE

INCLUSION OF AHRQ PRIORITY POPULATIONS: ACCEPTABLE

COMMITTEE BUDGET RECOMMENDATIONS: The budget was recommended as requested.
